# Supplementary material for: Epigenetic Ratchet: Spontaneous Adaptation via Stochastic Gene Expression
Source: Sci Rep. 2020 Jan 16;10:459. doi: 10.1038/s41598-019-57372-0 (PMC6965613; doi:10.1038/s41598-019-57372-0)
Supplement: Supplementary file 1 — Supplementary File. [file 41598_2019_57372_MOESM1_ESM.pdf]

Supplemental Text of "Epigenetic Ratchet: Spontaneous Adaptation via Stochastic Gene Expression"

Yusuke Himeoka and Kunihiko Kaneko

October 30, 2019

Contents

1 The stochastic equations for the model **1**
1.1 The master equation 1
1.2 The Fokker-Planck Equation 2
1.3 The Langevin Equation and the reduced Fokker–Planck Equation 3
1.4 The steady solution and the boundary in the σ–θ space 4

2 Modified Models **4**
2.1 The model with mRNA and a modifier gene or protein 4
2.2 Spontaneous deactivation of the epigenetic variable 5
2.3 Different values of the target protein concentration 8
2.4 Different function forms of growth rate 8
2.5 Different choices of the production rate of the target protein 9
2.6 Alternative choice of $H(P_s)$ 9
2.7 The chemical reaction network model 10

1 The stochastic equations for the model

1.1 The master equation

The time evolution of the probability of the state with N_{P_s}, N_{P_t}, and N_{E_t} of the sensor protein, the target protein, and the epigenetic modification level[1] at time t (written as

[1] while the "epigenetic modification level" could be different from the number of molecules, here we count the modification level as if it is the number of molecules.

$Q(t; N_{P_s}, N_{P_t}, N_{E_t}) = Q(t, \vec{N}))$ is governed by the master equation given as

$$\begin{aligned} \frac{\partial}{\partial t}Q(t,\vec{N}) &= v_s\Omega(\mathbb{E}_{P_s}^{-1}-1)Q(t,\vec{N}) + d_s(\mathbb{E}_{P_s}-1)N_{P_s}Q(t,\vec{N}) + (\mathbb{E}_{P_s}-1)\mu(N_{P_t}/\Omega)N_{P_s}Q(t,\vec{N}) \\ &+ v_tN_{E_t}(\mathbb{E}_{P_t}^{-1}-1)Q(t,\vec{N}) + d_t(\mathbb{E}_{P_t}-1)N_{P_t}Q(t,\vec{N}) + (\mathbb{E}_{P_t}-1)\mu(N_{P_t}/\Omega)N_{P_t}Q(t,\vec{N}) \\ &+ l_+H(N_{P_s}/\Omega)\Omega(\mathbb{E}_{E_t}^{-1}-1)Q(t,\vec{N}) + l_-H(N_{P_s}/\Omega)(\mathbb{E}_{E_t}-1)N_{E_t}Q(t,\vec{N}), \end{aligned} \tag{S1}$$

where Ω indicates the system size, and $\mathbb{E}_i$ is the step operator of chemical species i which acts to an arbitral function $f(\cdots, N_i, \cdots)$ as $\mathbb{E}_i f(\cdots, N_i, \cdots) = f(\cdots, N_i+1, \cdots)$. The growth rate and $H(\cdot)$ is defied as described in the main text, respectively

1.2 The Fokker-Planck Equation

The Kramers-Moyal (KM) expansion is applied to the master equation (S1) by regarding the number of molecules as continuous numbers. By truncating the KM expansion up to the second order in derivative, we get the Fokker-Planck equation as follows

$$\begin{aligned} \frac{\partial}{\partial t}Q(t,\vec{N}) &= -\frac{\partial}{\partial N_{P_s}}\Big(v_s\Omega - (d_s+\mu(N_{P_t}/\Omega))N_{P_s}\Big)Q(t,\vec{N}) \\ &- \frac{\partial}{\partial N_{P_t}}\Big(v_tN_{E_t} - (d_t+\mu(N_{P_t}/\Omega))N_{P_t}\Big)Q(t,\vec{N}) \\ &- \frac{\partial}{\partial N_{E_t}}\Big(H(P_s/\Omega)(l_+\Omega - l_-N_{E_t})\Big)Q(t,\vec{N}) \\ &+ \frac{1}{2}\frac{\partial^2}{\partial N_{P_s}^2}\Big(v_s\Omega + (d_s+\mu(N_{P_t}/\Omega))N_{P_s}\Big)Q(t,\vec{N}) \\ &+ \frac{1}{2}\frac{\partial^2}{\partial N_{P_t}^2}\Big(v_tN_{E_t} + (d_t+\mu(N_{P_t}/\Omega))N_{P_t}\Big)Q(t,\vec{N}) \\ &+ \frac{1}{2}\frac{\partial^2}{\partial N_{E_t}^2}\Big(H(P_s/\Omega)(l_+\Omega + l_-N_{E_t})\Big)Q(t,\vec{N}). \end{aligned}$$

Finally, we transform the variables as $N_{P_s} = \Omega P_s$, $N_{P_t} = \Omega P_t$, and $N_{E_t} = \Omega E_t$ leading to

$$\begin{aligned} \frac{\partial}{\partial t}Q(t,\vec{X}) &= -\frac{\partial}{\partial P_s}\Big(v_s - (d_s+\mu(P_t))P_s\Big)Q(t,\vec{X}) - \frac{\partial}{\partial P_t}\Big(v_tE_t - (d_t+\mu(P_t))P_t\Big)Q(t,\vec{X}) \\ &- \frac{\partial}{\partial E_t}\Big(H(P_s)(l_+ - l_-E_t)\Big)Q(t,\vec{X}) + \frac{\sigma^2}{2}\frac{\partial^2}{\partial P_s^2}\Big(v_s + (d_s+\mu(P_t))P_s\Big)Q(t,\vec{X}) \\ &+ \frac{\sigma^2}{2}\frac{\partial^2}{\partial P_t^2}\Big(v_tE_t + (d_t+\mu(P_t))P_t\Big)Q(t,\vec{X}) + \frac{\sigma^2}{2}\frac{\partial^2}{\partial E_t^2}\Big(H(P_s)(l_+ + l_-E_t)\Big)Q(t,\vec{X}), \end{aligned}$$

where $\vec{X}$ is the state vector $\vec{X} = (P_s, P_t, E_t)$, and the noise amplitude σ is defined as $\sigma^2 = 1/\Omega$. The drift term for each variable corresponds to the deterministic equation shown in the main text (Eq.(1)-(3)).

1.3 The Langevin Equation and the reduced Fokker–Planck Equation

From the Fokker Planck equation which is shown above, we can obtain the Langevin equation for each variable with the Ito interpretation given as

$$\frac{dP_s}{dt} = v_s - d_sP_s - \mu P_s + \sigma\sqrt{v_s + d_sP_s + \mu P_s}\xi_{P_s}(t) \tag{S2}$$

$$\frac{dP_t}{dt} = v_tE_t - d_tP_t - \mu P_t + \sigma\sqrt{v_tE_t + d_tP_t + \mu P_t}\xi_{P_t}(t) \tag{S3}$$

$$\frac{dE_t}{dt} = H(l_+ - l_-E_t) + \sigma\sqrt{H(l_+ + l_-E_t)}\xi_{E_t}(t), \tag{S4}$$

where $\xi_i(t)$ is Gaussian white noise which satisfies $< \xi_i(t)\xi_j(t) >= \delta_{ij}\delta(t-t')$ with δ_{ij} as the Kronecker's delta and $\delta(t)$ as the Dirac's delta function. Now, to make the calculation easy, we set $d_t \gg \mu_{\max}$ in additon to $\mu_{\max} \gg d_s$.

To derive a single-variable Fokker–Planck equation, we transform $l_\pm$ and t as $l_\pm = \epsilon k_\pm$ and $\tau = \epsilon t$ with ϵ as the smallest parameter among all the parameters appeared in Eq.(S2)-(S4). Then, the Langevin equations are rewritten as

$$\frac{dP_s}{d\tau} = \epsilon^{-1}\Big(v_s - d_sP_s - \mu P_s\Big) + \frac{\sigma}{\sqrt{\epsilon}}\sqrt{v_s + d_sP_s + \mu P_s}\xi_{P_s}(\tau) \tag{S5}$$

$$\frac{dP_t}{d\tau} = \epsilon^{-1}\Big(v_tE_t - d_tP_t\Big) + \frac{\sigma}{\sqrt{\epsilon}}\sqrt{v_tE_t + d_tP_t}\xi_{P_t}(\tau) \tag{S6}$$

$$\frac{dE_t}{d\tau} = H(k_+ - k_-E_t) + \sigma\sqrt{H(k_+ + k_-E_t)}\xi_{E_t}(\tau), \tag{S7}$$

where $\xi_i(\tau)$ satisfies $< \xi_i(\tau)\xi_j(\tau') >= \delta_{ij}\delta(\tau-\tau')$. We suppose ϵ is sufficiently small so that $\epsilon^{-1} \gg \sigma\epsilon^{-1/2}$ holds, and ignore the $\epsilon^{-1/2}$ terms appeared in Eq.(S5) and (S6) [1–3]. This assumption also means P_s and P_t are the fast variables comparing E_t, and thus, we can substitute the steady solutions of P_s and P_t which are obtained from the deterministic limit of Eq.(S5) and (S6) as functions of E_t into Eq.(S7) leading to

$$\frac{dE_t}{d\tau} = \tilde{H}(E_t)(k_+ - k_-E_t) + \sigma\sqrt{\tilde{H}(E_t)(k_+ + k_-E_t)}\xi_{E_t}(\tau). \tag{S8}$$

$$\tilde{H}(E_t) = \frac{1}{1 + \exp[-\tilde{\beta}((E_t - E_t^*)^2 - \tilde{\theta})]} \tag{S9}$$

where $\tilde{\beta} = \beta \times (v_t/d_t)^2 \times v_s/\mu_{\max}$, $\tilde{\theta} = (\theta \times \mu_{\max}/v_s - 1) \times (d_t/v_t)^2$, and $E_t^* = P_t^* \times d_t/v_t$. By constructing the Fokker–Plack equation from Eq.(S8), we get the equation

$$\begin{aligned}\frac{\partial}{\partial t}F(E_t,t) &= -\frac{\partial}{\partial E_t}\tilde{H}(E_t)(l_+ - l_-E_t)F(E_t,t) \\ &+ \frac{\sigma^2}{2}\frac{\partial^2}{\partial E_t^2}\tilde{H}(E_t)(l_+ + l_-E_t)F(E_t,t),\end{aligned} \tag{S10}$$

where $F(E_t, t)$ is the probability density function that the epigenetic variable has the value E_t at time t. The above equation agrees with Eq.(4) in the main text.

1.4 The steady solution and the boundary in the σ–θ space

The steady solution of Eq.(S10) is given as

$$F_{\rm st}(E_t) = \frac{1}{Z}(l_+/l_- + E_t)^{\nu-1}e^{-2E_t/\sigma^2}/\tilde{H}(E_t)$$

with Z as the normalization factor and $\nu = \frac{4l_+}{l_-\sigma^2}$.
The boundary which separates the adaptive regime (the average growth rate is close to $\mu_{\max}$) and non-adaptive regime ($< \mu >$ is close to zero) is calculated by setting $F_{\rm st}(E_t = l_+/l_-) = F_{\rm st}(E_t = E_t^*)$. It results in

$$\sigma^2 = \frac{2l_+}{l_-}\left[\ln\left(\frac{1+e^{-\tilde{\beta}\tilde{\theta}}}{e^{-\tilde{\beta}\tilde{\theta}}+e^{-\tilde{\beta}\delta^2}}\right) - \ln\left(\frac{2}{1+\gamma}\right)\right]^{-1}\left[2\ln\left(\frac{2}{1+\gamma}\right)+\gamma-1\right], \tag{S11}$$

where γ and δ is the ratio and difference between the two E_t values given by $\gamma = E_t^*/(l_+/l_-)$ and $\delta = E_t^* - l_+/l_-$, respectively. By assuming that E_t^* is sufficiently lsarger than l_+/l_- (specifically, $\gamma \gg 1$ and $\delta \gg 1/\tilde{\beta}$), Eq.(S11) is approximated as

$$\sigma^2 \approx \frac{2l_+}{l_-}\frac{\gamma - 2\ln(\gamma/2)}{\tilde{\beta}\tilde{\theta} + \ln(\gamma/2)}$$

which shows the reciprocal relationship between σ and $\tilde{\beta}\tilde{\theta}$.

2 Modified Models

2.1 The model with mRNA and a modifier gene or protein

Although we ignored the dynamics of mRNA concentration and modifier genes and proteins in the main text, this simplification is not crucial to obtain the main results. To see this, we introduce an extended model which consists of the concentration of mRNA (R_*) and protein (P_*) for sensor (s), modifier (m), and target (t) genes. The epigenetic variable is considered only for the target gene as in the main text. The corresponding genes are transcribed into mRNAs, and corresponding proteins are translated from the mRNAs. Both mRNAs and proteins have the degradation and dilution terms. Schematically, the interactions among the three genes or proteins are represented as Sensor $\xrightarrow{\pm}$ Modifier $\xrightarrow{\pm}$

Target, where the positive (negative) sign indicates the activation (inhibition). Via the rate equations, the dynamics of the above model are given as

$$\begin{aligned}\frac{dE_t}{dt} &= l_+ P_m - l_- P_m E_t, \\ \frac{dR_n}{dt} &= J_n - \phi_n R_n - \mu R_n, (n = s, m, t) \\ \frac{dP_n}{dt} &= u_n R_n - d_n P_n - \mu P_n, (n = s, m, t)\end{aligned} \tag{S12}$$

with

$$J_s = v_s(\text{const.}), J_m = v_m H(P_s), J_t = v_t E_t, \tag{S13}$$

where ϕ_n and u_n denote the spontaneous degradation rate and translation rate of mRNA n, respectively, whereas the definitions for other parameters are the same as those in the main text.

Fig.S1 shows (a) time courses, (b) the average growth rate as a function of θ, (c) relative frequency of a modification event, and (d) the average growth rate as a function of the noise level. Although the shapes of functions shown in panels (b)–(d) are different from those in Fig. 1 in the main text, it is seen that the adaptation mechanism works.

2.2 Spontaneous deactivation of the epigenetic variable

In the main text, we ignored spontaneous changes in the epigenetic state because such changes occur slowly. Here, we introduce spontaneous deactivation of the epigenetic state of the target gene as an example of spontaneous changes. Then, the model equation of the epigenetic state is slightly modified as

$$\frac{dE_t}{dt} = l_+ H(P_s) - l_- H(P_s) E_t - \phi E_t, \tag{S14}$$

with ϕ as the rate of spontaneous deactivation. The equations for the remaining variables (P_s and P_t) are not altered in comparison with Eq.(1)-(3) in the main text. We derived the chemical Langevin equation and the corresponding Fokker–Planck equation for E_t as we did in former part of the supplement, accordingly[2]. The averaged growth rate computed from the solution of the FP equation is plotted in Fig. S2 as a function of spontaneous deactivation rate ϕ and θ. As shown in the figure, the averaged growth rate still keeps a higher value than that at the steady state of the model up to $\phi \approx l_\pm$.

[2] To derive the reduced FP equation for E_t, we assume that the ϕ is also written with the smallest parameter ϵ as $\phi = \epsilon\psi$.

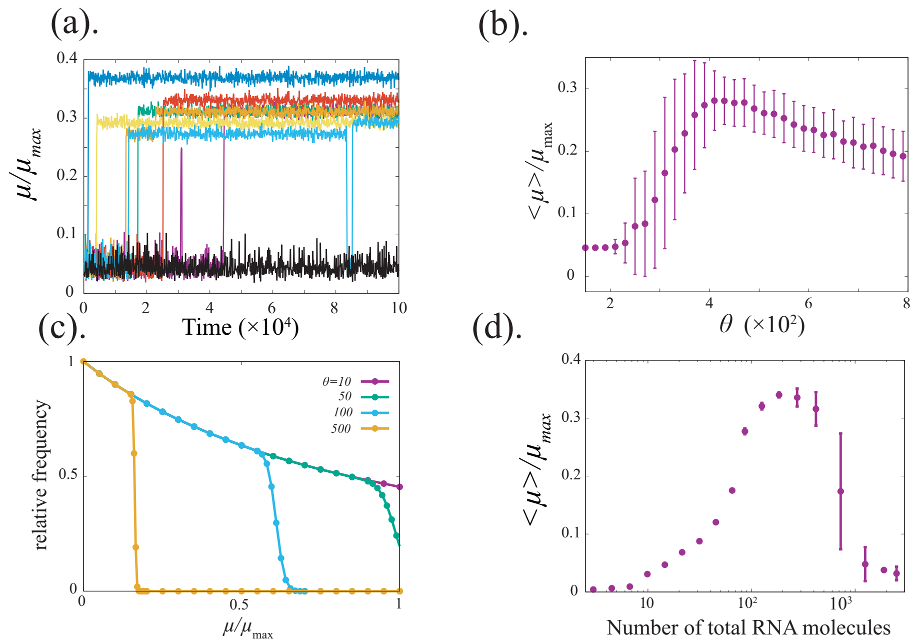

Figure S1: Adaptation via stochastic reactions. (a) Time courses of the growth rate relative to its maximum value for $\theta = 0$ and 400. While the growth rate fails to increase for the case with $\theta = 0$, all the 5 time courses of the growth rate μ for $\theta = 400$ exhibit the sequential increase. (b) The average growth rate as a function of θ. Each point was computed by averaging 100 samples of the time-averaged growth rate. The error bar indicates the standard error over the samples. The samples were obtained by simulations with different seeds of random numbers over randomly generated different initial conditions, whereas E_t and P_t are always set to zero initially. The growth rate is averaged from $t = 10^5$ to $t = 10^6$. (c) Relative frequency of modification events plotted against the growth rate μ/μ_{max}. The relative frequency is obtained by dividing the number of events at μ by that at $\mu = 0$. Data from different values of θ are plotted with different colors. The frequency is obtained for a given growth rate at the moment. For all cases, the frequency of the modification decreases as the growth rate increases, and the decrease is steeper for larger θ. (d) Dependence of the average growth rate on the number of molecules in the reaction system. The average growth rate increases when the number of molecule is intermediate i.e., at the moderate level of noise. Parameters are set as $u_i = v_i = 10, \phi_i = 1, (i = s, m, t), d_s = 10^{-2}, d_m = d_t = 10, l_p = l_m = 10^{-2}, \beta = 5, P_t^* = 15, \mu_{\max} = 1$, and $\Omega = 25.0$.

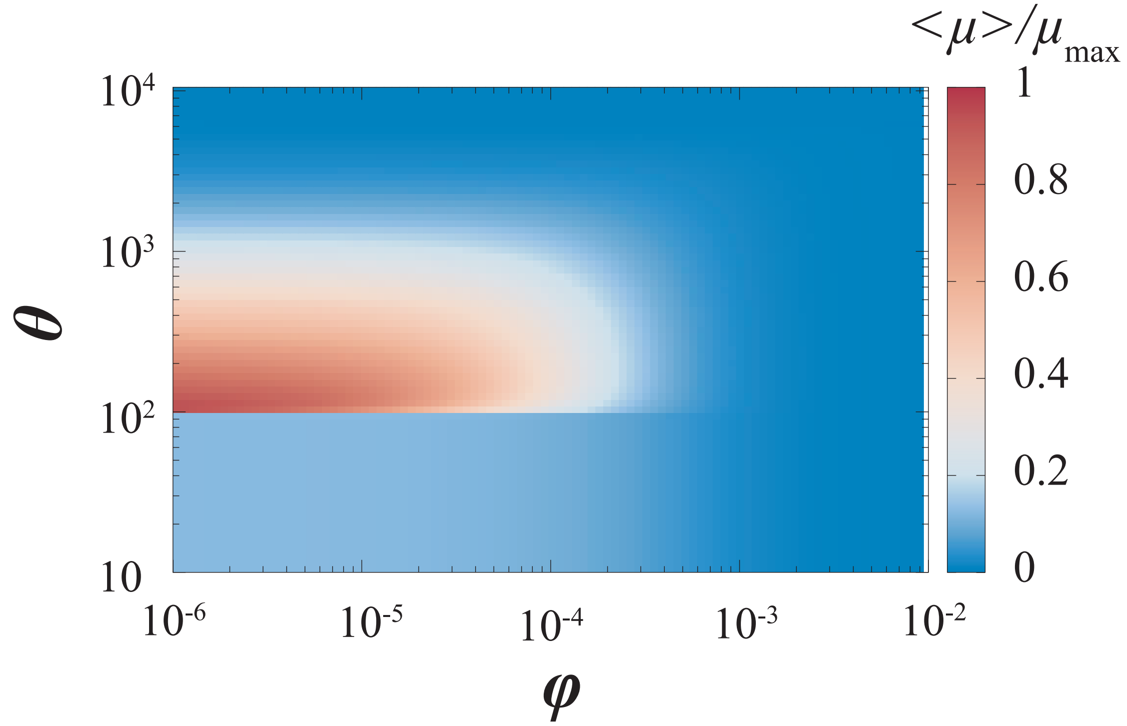

Figure S2: The averaged growth rate is plotted as a function of the spontaneous deactivation rate ϕ and the threshold θ. σ is 0.5, whereas other parameter values are chosen to be the same as those in Fig. 2 in the main text.

2.3 Different values of the target protein concentration

Robustness of the adaptation mechanism is examined by computing the average growth rate based on the solution of the Fokker–Planck equation (Eq.(S11)) for different values of E_t^* (i.e., P_t^*). Fig. S3 shows the average growth rate as a function of E_t^* and θ. It can be seen that the growth rate is also enhanced over different target values E_t^*, as long as θ value is sufficiently large. The boundary in (E_t^*, θ) space (Eq.(S11)) which separates the adaptive and non-adaptive regime is also drawn.

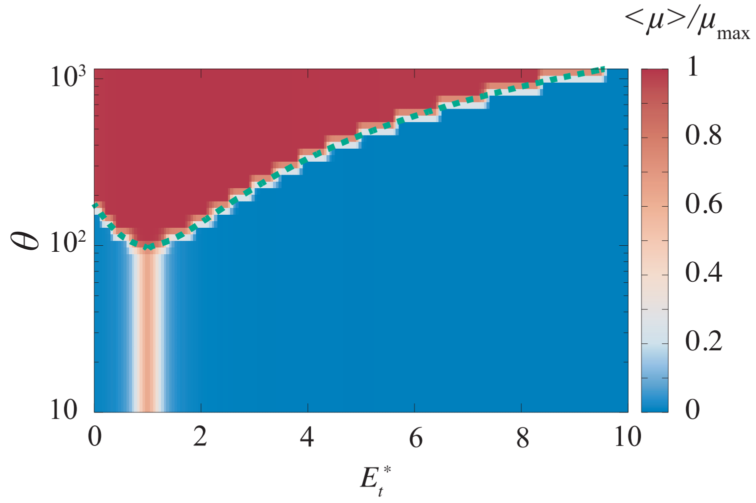

Figure S3: The averaged growth rate is plotted as a function of E_t^* and θ. The growth rate has a high value around $E_t^* = 1$ regardless of θ values because $E_t^* = 1$ corresponds to the attractor value of the deterministic model. The green dashed line indicates the boundary which separates the adaptive and non-adaptive regime. σ is set as 0.1, and the same values in Fig. 2 in the main text are chosen for the other parameters.

2.4 Different function forms of growth rate

In the main text, we chose $\mu = \mu_{\max}/(1 + (P_t - P_t^*)^2)$ as the growth rate. To demonstrate that the results shown in the main text are not altered qualitatively even if the different forms of the growth rate function are adopted, we computed the averaged growth rate for two different examples of growth rates: $\mu = \frac{\mu_{\max} P_t}{K + P_t}$ and $\mu = \frac{\mu_{\max}}{1 + P_t}$. Fig. S4 shows the averaged growth rate obtained from the Fokker–Planck equation with the respective growth rate function. In both cases, the averaged growth rate increases from the steady

growth rate of the original state without adaptation [3].

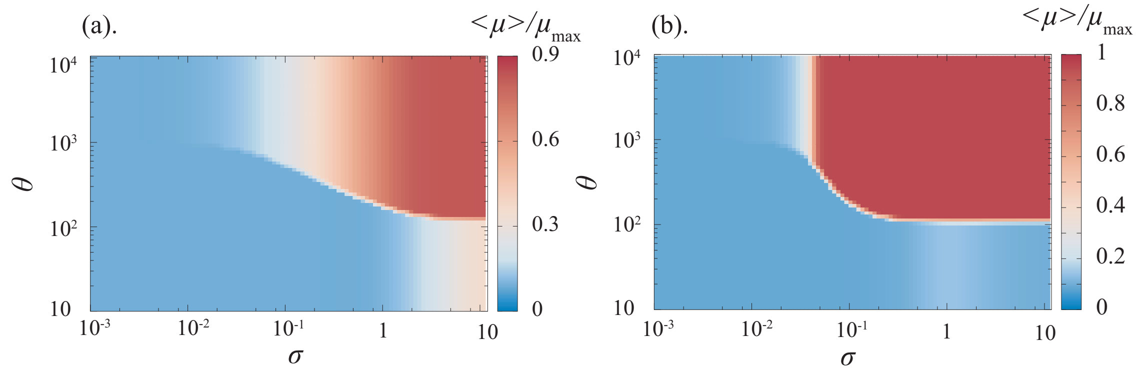

Figure S4: The averaged growth rate for two different definitions of growth rate μ. (a) The Michaelis-Menten type of function of P_t: $\mu = \frac{\mu_{\max} P_t}{K+P_t}$. (b).The inverse of P_t: $\mu = \frac{\mu_{\max}}{1+P_t}$. 1 in the denominator is added to prevent the divergence of the growth rate at $P_t = 0$. K is set to be 100. The same parameter values as in Fig.2 in the main text are chosen for the other parameters.

2.5 Different choices of the production rate of the target protein

In the main text, we chose $v_t E_t$ as the production rate of P_t. Here, we show that the production rate need neither to be proportional to E_t nor even to be an increasing function of E_t. We computed the averaged growth rate for two different examples of the production rate of the target protein growth rates: $\frac{v_t}{1+E_t}$ and $\frac{v_t E_t}{1+E_t}$. Fig. S5 shows the averaged growth rate obtained from the Fokker–Planck equation with the respective production rate of the target protein. In both cases, the averaged growth rate increases from the steady growth rate of the original state without adaptation.

2.6 Alternative choice of $H(P_s)$

While we adopted $H(P_s) = (1 + \exp[-\beta(P_s - \theta)])^{-1}$ in the present study just for reducing computation time, here we show the adaptation mechanism works with $H(P_s)$ as a Hill

[3]For Fig. S4(a), however, the averaged growth rate increases in the large noise region regardless of the value of $\tilde{\theta}$. This should be seen as an artifact of the definition of the growth rate. The greater the noise amplitude, the broader the distribution of E_t is. Given that the growth rate monotonically increases with the concentration of the target protein, the growth rate increases if the distribution of E_t becomes broad. Thus, the growth rate monotonically increases against the noise amplitude σ, regardless of the value of θ.

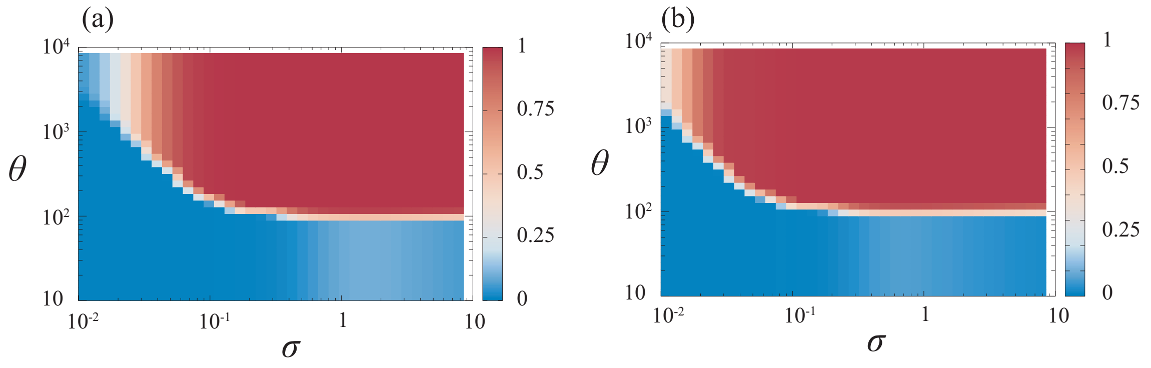

Figure S5: The average growth rate for two different definitions of the target protein production rate. (a) Decreasing function of E_t, as given by $\frac{v_t}{1+E_t}$. (b). Michelis-Menten-type function as given by $\frac{v_t E_t}{1+E_t}$, where 1 in the denominator is added to prevent the divergence of the growth rate at $E_t = 0$. v_t is set to be 500 so that P_t cannot reach P_t^* with the default v_t value (v_t=100). For the other parameters, the same values as in Fig.2 in the main text are adopted.

function given as $H(P_s) = P_s^{n_H}/(\theta^{n_H} + P_s^{n_H})$ with n_H as the Hill coefficient. In Fig.S6, we plotted the growth rate averaged by the probability distribution function given in Eq.(S11) with $H(P_s)$ as the Hill function instead of the original choice. The choice of $H(P_s)$ does not alter the result qualitatively as it keeps a threshold-type shape.

2.7 The chemical reaction network model

In the main text, we studied the model with only one target gene–protein pair, for simplicity. In general, however, the number of target gene-protein pairs can be more than one. Thus, we constructed a model by combining the sensor–target system in the main text with the phenomenological metabolic reaction networks having many components. For the metabolic reactions, we have N nutrient species, G growth factors, and $M - N - G$ intermediate metabolites. The model cell takes up nutrients from an external environment and converts them into growth factors via several metabolic reactions. The specific growth rate of the cell is determined by the concentration of the growth factors, accordingly.
We have multiple target gene–protein pairs in this model which have different steady-state concentrations for each, and each target protein serves as a catalyst for metabolic and nutrient uptake reactions. The number of sensor proteins is set to one. Instead of introducing multiple sensors, we set different affinity values of the epigenetic state for the modification. Finally, the dilution effect for substrates is ignored because the timescale of metabolic reactions including uptake reactions is typically much shorter relative to the growth rate.

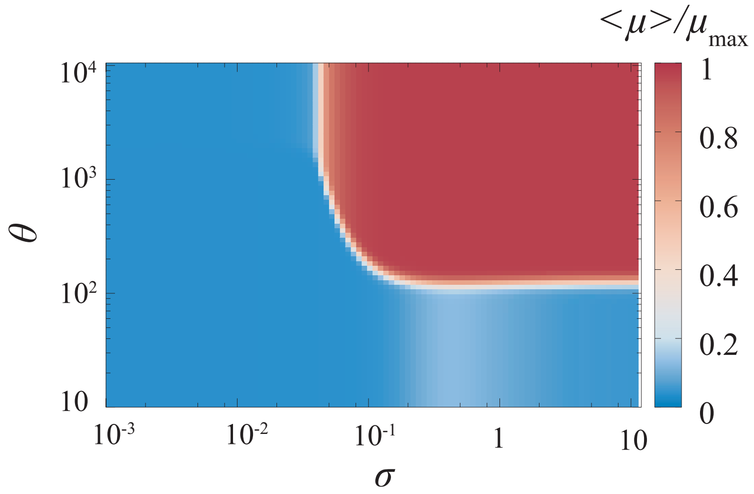

Figure S6: The average growth rate in the case by adopting a Hill function for $H(P_s)$ is plotted as a function of θ and σ. The Hill coefficient n_H is used for determining the steepness of the function instead of β. n_H is set as 20, whereas the same parameter values as in Fig.2 in the main text are chosen for the remaining parameters.

The model equation for the sensor–target system is thus given by

$$\begin{aligned} \frac{dE_t^n}{dt} &= c_n H(P_s)(l_+^n - l_- E_t^n), \\ \frac{dP_s}{dt} &= v_s - d_s P_n - \mu P_s, \\ \frac{dP_t^n}{dt} &= v_t E_t^n - d_t P_t^n - \mu P_t^n, \end{aligned} \tag{S15}$$

where l_+^ns are different among n while l_- takes a common value, and c_n represents the affinity of the nth target gene for the sensor protein. We use the mass–action kinetics for the metabolic reaction and set the rate constants of metabolic reactions to unity by assuming that the difference in the rate constant is reflected in the heterogeneity of l_+^n (i.e., the rate of a chemical reaction is the simply a product of the concentrations of the substrate and the target protein that catalyzes the reaction). Then, the model equation for the metabolites is expressed as follows:

$$\frac{d\vec{S}}{dt} = C(\vec{P}_t) \cdot \vec{S} - d \cdot I \cdot \vec{S} + \vec{U}(\vec{P}_t), \tag{S16}$$

where C is the matrix representing the reaction rate of a chemical reaction per unit amount of the substrate. If the reaction from the ith metabolite to the jth metabolite exists, one

target protein of the reaction (indexed here as k) is allocated and C_{ij} and C_{ji} are assigned as P_t^k and $-P_t^k$, respectively. If there is no reaction between i and j, then C_{ij} and C_{ji} are set to zero. $d \cdot I \cdot \vec{S}$ represents the spontaneous degradation at the uniform rate d with I as the unit matrix, whereas $\vec{U}(\vec{P}_t)$ indicates the uptake reactions. The uptake reactions are assigned only to the nutrient chemicals. For each uptake reaction, one target protein is assigned to a transporter protein (indexed as j here), and the uptake rate of the ith chemical species is represented as $U_i = P_t^j \cdot S_{\text{ext}}^i$ with the external concentration of the ith chemical S_{ext}^i. The growth rate is defined as $\mu = \mu_{\max} \frac{S_{\min}}{K + S_{\min}}$, with $S_{\min}$ as the minimum concentration among G growth factors and a constant, K.
We performed stochastic simulations of the model (S15) using the Gillespie algorithm[4], while we solved the deterministic equation for the metabolites (S16) at every update of (P_s, E_t^n, P_t^n) because the differences in the number of molecules and timescales between metabolic reactions and protein synthesis are quite large. The average growth rates are shown in Fig.3 in the main text.

References

[1] Hannes Risken. Fokker-planck equation. In *The Fokker-Planck Equation*, pages 63–95. Springer, 1996.

[2] Hermann Haken. *Synergetics: Introduction and advanced topics.* Springer Science & Business Media, 2013.

[3] Kunihiko Kaneko. Adiabatic elimination by the eigenfunction expansion method. *Progress of Theoretical Physics*, 66(1):129–142, 1981.

[4] First, we generate connected and directed random networks with $N = 1, G = 4$, and $M = 16$. Then, we selected networks to verify that the reaction network can achieve both lower and higher growth rates by tuning the expression level of the target genes. To be specific, we randomly generated 100 sets of $\mathbf{P_{t,k}} = (P_{t,k}^0, \cdots, P_{t,k}^{R-1}), (0 \leq k < 100)$ values (R is the total number of metabolic reactions that depend on the network configuration), and computed steady growth rate μ_k^{st} for each $\mathbf{P_{t,k}}$ sample. If the minimum value of μ_k^{st} is below 0.1, and the maximum value is above 0.75, then we choose the network, and set l_+^ns so that the minimum growth rate is realized in the steady state without noise. Parameter values are chosen to be $S_{\text{ext}} = 1, d = 1, K = 0.1, \beta = 5, l_m = 10^{-2}, v_t = 100, v_s = 1, d_s = 10^{-2}, d_t = 100, \mu_{\max} = 1$, and $\Omega = 1$. Affinity c_n is assigned by generating uniform random numbers within $(0, 1)$, and l_+^n is assigned by generating uniform random numbers within $(1, 10)$.
